# Past, present, and future perspectives of biodegradable films for soil: A 30-year systematic review

**DOI:** 10.3389/fbioe.2022.1006388

**Published:** 2022-10-17

**Authors:** Yitao Sun, Wenlong Yang, Hongxia Shi, Sikander Khan Tanveer, Jiangbo Hai

**Affiliations:** ^1^ College of Agronomy, Northwest A&F University, Yangling, China; ^2^ National Agriculture Research Center, Islamabad, Pakistan

**Keywords:** biodegradable film, soil, quantitative analysis, hot frontiers, knowledge structure

## Abstract

Based on the Web of Science Core Collection (WOSCC) database, the academic works published in the past 30 years on biodegradable films for soil were analyzed. In order to ensure the rigor of this experiment, this paper is based on the mathematical double matrix model VOS Viewer software and CiteSpace software. This work shows that publications of biodegradable films for soil are increasing year by year; polymer science is the hottest subject in the field of biodegradable films for soil; China and the United States are the countries with the most significant number of publications in this field, has an important position; Washington State University is the most published institution. This study further identifies and reveals the essential characteristics, research strength, knowledge structure, main research fields, and research hotspots in the late stage of the field of biodegradable films for soil and introduces the Activity Index (AI) and the Attractive Index (AAI), thereby assessing trends and performance in different countries. The paper also further illustrates the importance of biodegradable films by presenting field trials using biodegradable films on different plants. The research in the field of biodegradable films for soil is divided into four categories: “The research field of degradation,” “The effect of biodegradable film on soil,” “Performance and mechanism of the biodegradable film,” and “Effects of biodegradable film on crop growth and development.”. The study can be seen as a microcosm of the development of biodegradable films for soils, which will help researchers quickly identify their general patterns. Readers can better understand the changes and development trends in this field in the past 30 years and provide references for future research.

## 1 Introduction

With the continuous improvement of the level of global industrialization and urbanization, the world today is undergoing tremendous changes unseen in a century, and the ensuing energy crisis and environmental problems have attracted significant attention from leaders of various countries (Titirici et al., 2015; Luo et al., 2021; Zhang et al., 2021). Soil is a fragile, non-renewable resource whose health and sustainable development are affected by human activities and natural processes (Bouma, 2014; Keesstra et al., 2016). Plastic film is a material applied to or grown on the soil surface (Liang et al., 2002). At the same time, due to the lack of biodegradability of plastic itself and the massive and inappropriate use of human beings, more and more plastic wastes are found in natural ecosystems such as soil, wetlands, rivers, and oceans, bringing a substantial environmental impact to the earth and ecological crisis (Barboza et al., 2018; Deng et al., 2019).

When the soil is covered with film, it will promote the growth and development of annual and perennial crops and increase the yield (Schonbeck and Evanylo, 1998; Weber, 2003; Liu et al., 2014). Today, the most common film for soil is plastic film. Plastic film can effectively improve the field microclimate for crop growth, increase crop yield, and enable crops to develop well in soils with limited moisture (Kader et al., 2017; Lamont, 2005; R. Li R et al., 2021). However, the residues of plastic film will seriously harm the ecological environment and have a series of adverse effects on the soil, such as reducing soil porosity, soil water content, and increasing soil bulk density (Y. L. Qi Y. L et al., 2020). Therefore, to ensure the sustainability of the ecosystem, protect the ecological environment, and alleviate the energy crisis, the development and use of new film materials have become a new trend in the research progress of soil film. To date, various films have been developed that can be degraded by microorganisms in the soil, including paper films (Siwek et al., 2019), oxidatively degradable plastic films (Nagashima et al., 2012), and biodegradable films (Hayes et al., 2017; Saglam et al., 2017; Serrano-Ruiz et al., 2018). Biodegradable materials that can be degraded by microorganisms (bacteria, fungi, or algae), either from renewable or fossil fuel resources or a mixture of both (Vroman and Tighzert, 2009). The products of this decomposition are water, carbon dioxide, or methane (only under anaerobic conditions), with no residues or new organisms (Orgiles-Calpena et al., 2014; Siwek et al., 2019). The biodegradable film is innovative biotechnology designed to maintain traditional plastic film production properties (Siwek et al., 2019). American Society for Testing and Materials (ASTM) (Gueymard et al., 2002) defines the biodegradable film as a material that can degrade from natural microorganisms such as bacteria, fungi, and algae.

Over the past three decades, numerous academic papers have been published on biodegradable films for soil. However, these substantial scientific achievements are not conducive to our quick grasp of hot spots and insight into future directions. To more fully illustrate the importance of biodegradable films to soils, rigorous quantitative analysis and statistical justification based on mathematical models are required. A significant disadvantage of conducting retrospective studies under traditional methods is the reliance on limited publications to create personal biases that can lead to biased and flawed results. In contrast, bibliometric analysis overcomes the subjective factors in traditional reviews. It can quantitatively explore knowledge structures, research hotspots, and insights into specific scientific fields by comprehensively filming all literature in a selected period and omitting crucial literature. New Insights (Yeo et al., 2014). Bibliometric analysis is a new data-driven method that applies statistical methods to scientific results and is widely used in research trend detection, institutional cooperation analysis, national cooperation analysis, changes in subject areas, etc., with knowledge-oriented quantitative functions (Kapanen et al., 2008; Y. Q. Sun et al., 2019; Tan et al., 2021; Zhou et al., 2018). By filtering and processing a large amount of information, the correlation between various data can tap the potential value of knowledge. As mathematics, statistics, and computer science underlie its discipline, bibliometrics can provide intuitive data analysis and accurate insights into the progress of scientific research (Durieux and Gevenois, 2010; Bornmann and Mutz, 2015). The purpose of this paper is to perform bibliometric analysis using the analysis tools that come with Web of Science (WOS), VOS Viewer software, and CiteSpace software (C. M. Chen, 2006; van Eck and Waltman, 2010), and a comprehensive review and in-depth assessment of the research field of biodegradable films for soils, with unique insights into its prospects and opportunities, shed light on current research hotspots and knowledge gaps.

This study is mainly carried out from four perspectives: 1) Understanding the research strength of biodegradable films for soil (publication trends, representative countries, institutions, essential subject areas, citations, and representative journals); 2) Recognize the knowledge structure of this research field; 3) Reveal the changing trend of research topics and research hotspots; 4) Determine future research directions in this field.

## 2 Methodology and data

### 2.1 Data source and retrieval

The critical research work of this study is determined to be 30 years from 1992 to 2021, and related articles on biodegradable films for soil are carried out in the Web of Science Core Collection (ScienceCitation Index Expanded, SCIE) database, and the retrieval time is 2022 April 24. The reason why the WOSCC database is selected for search is that compared with other databases (PubMed and Scopus), it contains more than 10,000 subject fields such as environment, medical care, ecology, agriculture, etc., and has international authority, great influence, high quality and long history of research data (Harzing and Alakangas, 2016; Mongeon and Paul-Hus, 2016; Zhao, 2017). In order to avoid bias due to the daily update of the WOS database, the articles required for the search were conducted within 24 April 2022, and articles published from 1 January 2022, onwards were excluded because, from this period, Any collection from that year will include incomplete bibliometric data for that year. The retrieval parameters for biodegradable membranes for soil are set as (TI = ((“biodegradable*” OR “biological degradation*” OR″biodegradation*” OR″bio degradable*” OR “biodegradability*”) and (“ film*” OR “films*” OR “ film film*” OR” film films*” OR “ film*” OR “ films*” OR “cover*”) and (“soil*” OR “land*” OR “farmland*” OR “cropland*” OR “cultivated land*”))) OR (AB = ((“biodegradable*” OR “biological degradation*” OR″biodegradation*” OR“bio degradable*” OR “biodegradability*”) and (“ film*” OR “films*” OR “ film film*” OR” film films*” OR ″ film*” OR ″ films*”OR"cover*”) and (“soil*” OR “land*” OR “farmland*” OR “cropland*” OR “cultivated land*”))), Search time range: (Time = 1 January 1992—31 December 2021).

The raw data analyzed in this paper is the content of 1222 publications in the field of biodegradable films for soils obtained from the Web of Science Core Collection (ScienceCitation Index Expanded, SCIE) database. Document types include 1086 research papers, 56 proceeding papers, 62 review papers, and 18 other types (meeting abstract, early access, editorial material, and note). These papers come from 200 institutions in 87 countries, covering 90 research directions, 200 published journals, and 200 authors. Most categories belong to the literature subject categories: Polymer Science, Environmental Sciences, Engineering Environmental, Agronomy, and Biotechnology Applied Microbiology.

### 2.2 Scientometric analysis methods

Referring to previous studies, for example, development trends and hotspots of global forest carbon sinks and natural disaster research (Huang et al., 2020; S. Shen et al., 2018), we employed two indexes, the Activity Index (AI) and the Attractive Index (AAI), to evaluate the relative effort to a research field and the relative impact made by a country in terms of citations of its publications. The AI indicates the relative effort a country puts into a research area, while the AAI shows the relative impact a country has had in attracting citations through its publications.

Activity Index (AI):

$${AI}_{i}^{t}=\frac{P_{i}^{t}/\sum P}{{TP}^{t}/\sum TP}$$ (1)

Attractive Index (AAI):

$${AAI}_{i}^{t}=\frac{C_{i}^{t}/\sum C}{{TC}^{t}/\sum TC}$$ (2)

$$Centrality\left(node\;i\right)=\underset{i\neq j\neq k}{\sum }\frac{P_{jk}\left(i\right)}{P_{jk}}$$ (3)

In Eqs 1, 2, ${AI}_{i}^{t}$ is the Activity Index of country i in year t; $P_{i}^{t}$ is the number of articles about a field published by country i in year t; ∑P is the total number of a field publications in a country during the publication period; ${TP}^{t}$ is year t of global a field publications; ∑TP is the sum of global a field publications over a period of time. Similarly, ${AAI}_{i}^{t}$ represents the attractiveness index of country i in year t; $C_{i}^{t}$ is the citations of a field publications in country i in year t; ∑C is the total number of citations of a field publications in country i over a period of time; ${TC}^{t}$ represents year t The global a field citations ∑TC in year t and t refers to the total number of a field citations in the same periodas as that of ∑C AI = 1 and AAI = 1 represent the average level of global a field research work and academic impact, respectively. AI>1 or AI<1 means a country’s research work is above or below the global average; AAI>1 or AAI<1 means a country attracts citations greater or less than the global average.

In Eq. 3, ρjk represents the number of shortest paths between node j and node k, and ρjk (i) is the number of those paths that pass-through node i. In CiteSpace, nodes whose betweenness centrality exceeds 0.1 are called key nodes. At the documental level, each document’s importance in a co-citing network can be partially evaluated by the indicator of betweenness centrality (Ghisi et al., 2020).

## 3 Results and discussion

### 3.1 Characteristics of publication outputs

**FIGURE 1:**
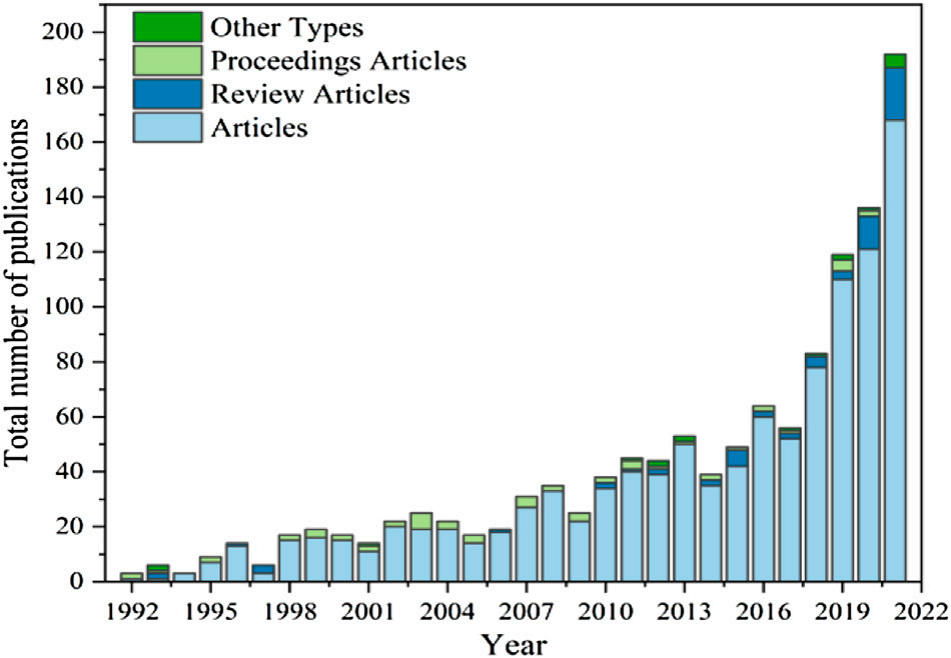
The distribution of the number of articles published from 1992 to 2021.

Figure 1 shows the number of articles published on biodegradable membranes used in soil from 1992 to 2021. The period from 1992 to 2000 was the initial stage, with 94 articles and an average annual publication of about 10.4. Although the number of articles is small, there are high-quality articles, such as Drumright, RE et al. (Drumright et al., 2000) in 1992, proving that polylactic acid (a biodegradable made of renewable resources, can produce H_2_O and CO_2_ after decomposition and hummus) can replace the plastics of petrochemical products and are used in many fields; 2001–2016 was a period of rapid growth, with a total of 542 publications and an average annual publication volume of about 33.9. The research in the field of the biodegradable membrane is more in-depth, and people’s research interest in this field is gradually increasing; 2017–2021 is an explosive growth stage, with a total of 586 papers published, with an average annual publication volume of about 117.2 papers, and in 2021 The number of publications reached its peak at 192.

### 3.2 Analysis of subject categories, countries, institutions, authors, and cited journals

#### 3.2.1 Subject categories

The top 10 subject categories in biodegradable films for soil are shown in Table 1, with a 10-year cycle divided into 1992–2001, 2002–2011, and 2012–2021. The field of biodegradable films for soil includes Polymer Science (365 papers, 29.87% of the total); Environmental Sciences (287, 23.49%); Engineering Environmental (173, 14.16%); Agronomy (87, 7.12%); Biotechnology Applied Microbiology (76, 6.22%); Chemistry Applied (75, 6.14%); Engineering Chemical (62, 5.07%); Water Resources (57, 4.66%); Soil Science (56, 4.58%) and Horticulture (53, 4.34%). It is worth noting that Polymer Science and Environmental Sciences fields accounted for more than 50% of the total number of articles, which shows that these two fields are the main research fields of biodegradable films for soil. From 2002 to 2011, publications in Agronomy, Chemistry Applied, and Horticulture increased significantly, with slow growth in other fields. It is worth noting that from 2012 to 2021, the publications in the fields of Agronomy, Chemistry Applied, and Horticulture still increased significantly, which shows that research in these three fields will still become the future research hotspots and trends.

**TABLE 1:** Article output in ten subject categories for research on biodegradable films for soil.

Subject category (biodegradable film for soil)	1992–2001	2002–2011	2012–2021
Polymer Science	41	107	217
Environmental Sciences	24	56	207
Engineering Environmental	19	44	110
Agronomy	2	13	72
Biotechnology Applied Microbiology	15	23	38
Chemistry Applied	3	12	60
Engineering Chemical	6	14	42
Water Resources	11	12	34
Soil Science	3	11	42
Horticulture	1	10	42

#### 3.2.2 Country

**FIGURE 2:**
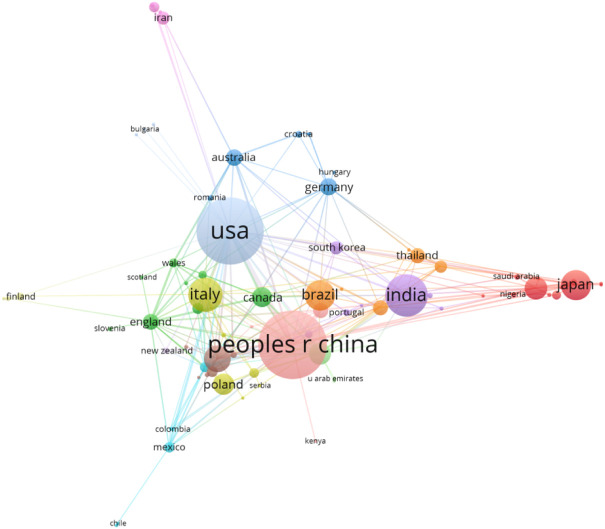
Knowledge map of cooperation between countr (freqiesuency ≥ 1).

To date, 87 countries worldwide have contributed to the field of degradable films for soil. Table 2 lists the top 10 most productive countries in this field. Visual analysis and mapping of study countries were performed using WOSCC database statistics and VOS Viewer software (Figure 2). Each node in Figure 2 represents a different country, the node’s size represents the scientific output of that country, and the colors and lines of the nodes represent different clusters based on the co-creation matrix of the corresponding country. The line between the two nodes represents the knowledge cooperation between countries, and the thickness of the line represents the degree of cooperation. The stronger the connection between the two nodes, the deeper the connection and cooperation between the two countries. The VOS Viewer software sets the parameter as the number of national publications greater than or equal to 1.

**TABLE 2:** Top ten most productive countries in terms of relevant articles.

Country (biodegradable film for soil)	Ps	Percentage (%)	TC^a^	TC/P^b^	h-index
China	231	18.90	4,592	19.88	32
United States	230	18.82	11,782	51.23	46
India	113	9.25	2,963	26.22	29
Italy	82	6.71	2,455	29.94	29
Brazil	71	5.81	1,085	15.28	19
Japan	69	5.65	1,684	24.41	24
Spain	56	4.58	1,844	32.93	23
France	44	3.60	1,726	39.23	22
Malaysia	44	3.60	679	15.43	15
Poland	40	3.27	577	14.43	13

Note: Ps: the total number of articles published. TC^a^: the total citations for a country. TC/P^b^: average number of citations per paper for a country. h-index: according to Hirsch in 2005 (Hirsch, 2005):A scientist has index H if H of his/her Np papers have at least H citations each, and the other Np-H papers have no more than H citations each, in which Np is the number of articles published during n years. A higher H-index indicates greater academic impact.

In the field of biodegradable films used in soil, China and the United States are the most representative countries, of which China has published 231 papers, accounting for 18.90% of the world; TC^a^ value is 4,592 times, TC/P^b^ value is 19.88, the h-index value is 32; followed by the United States (230, 18.82%, 11,782, 51.23, 46), the TC/P^b^ value of the United States is the highest among the top ten countries, at 51.23, and the h-index value is also the highest, at 46. Although the number of publications in the United States is not the highest among the top ten countries, the h-index calculated by the system is the highest due to the highest number of citations. It can be seen that the United States has tremendous academic influence. As shown in Figure 2, the United States and China have the most frequent cooperation, followed by the United States and India and China and India.

Currently, the United States uses about 450,000 tons of agricultural plastics every year, and China uses plastic films, with an estimated 12,500–140,000 tons of film applied annually, covering about 20 million hectares or 12% of China’s arable land (Liu et al., 2014; Tremblay, 2018). It can be seen that the United States and China have strong financial and policy support for the field of biodegradable films for soil. Of course, other countries have also made great efforts and contributions to the development of this field. However, there is still little cooperation between different countries, and more cross-country cooperation among scholars is needed in the future.

#### 3.2.3 Institution

**FIGURE 3:**
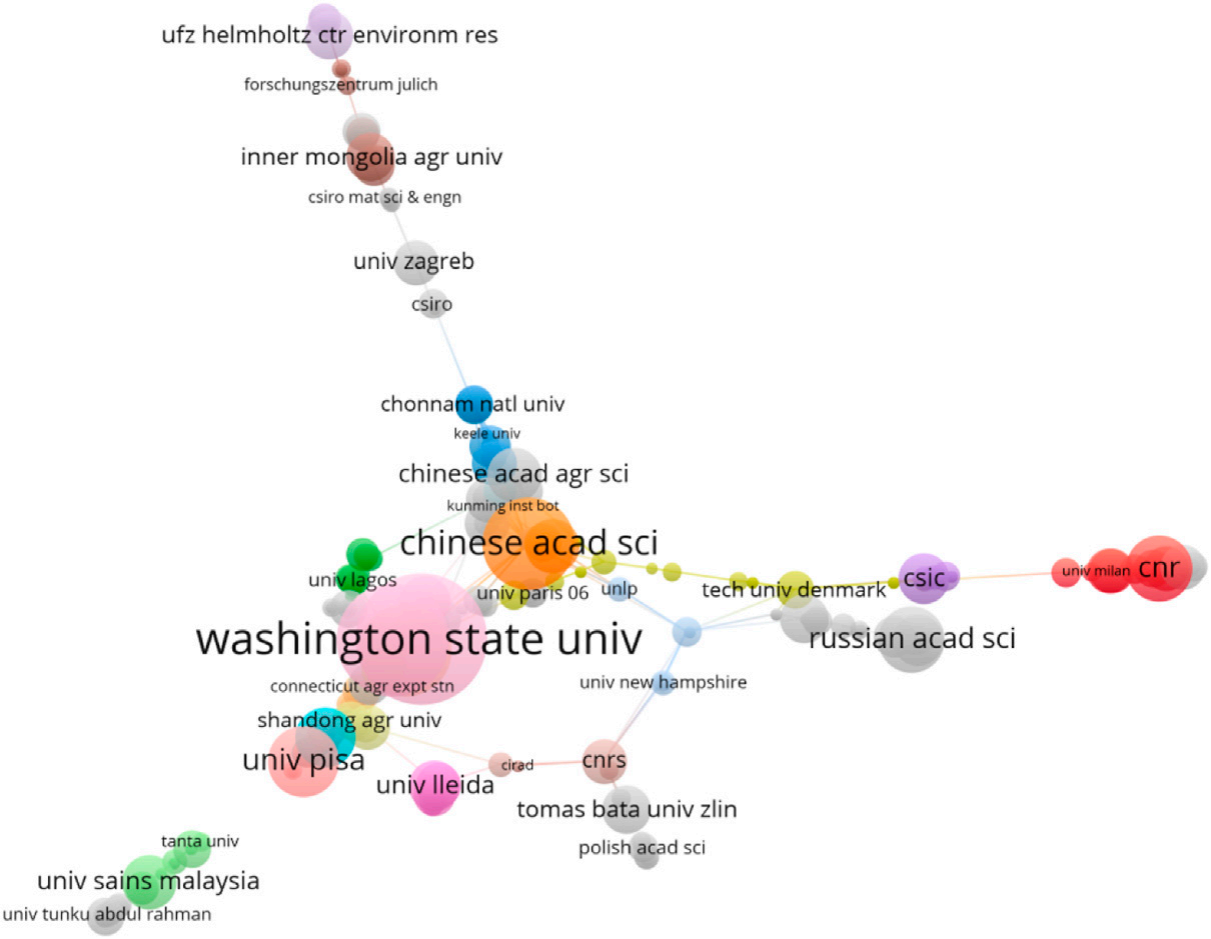
Knowledge map of cooperation between institutions (frequency ≥ 1).

In the same research field, cooperation between institutions can reflect the most productive organizational, professional information, and inter-institutional relationships, and the frequency analysis and bibliographic coupling analysis of institutions are done through bibliometrics. A total of 200 institutions worldwide have contributed to biodegradable films for soil. Table 4 summarizes the top ten institutions in this field regarding publication volume. To further explore the collaboration between institutions, taking into account the impact of citations on the results, the literature coupling method was compared with the co-occurrence analysis (Amirbagheri et al., 2019)can better demonstrate the complex relationship between elements, mine highly consistent unit clusters, and help scholars who are engaged in research in the same field to find potential cooperative units (Boyack and Klavans, 2010). Research institutions in this field conduct literature coupling analysis and mapping through VOS Viewer software. The setting parameters are that the number of papers published by the institution is greater than or equal to 1 (Figure 3).

In the field of biodegradable films for soil, the most representative institution is Washington State University in the United States, which has published 47 papers, accounting for 3.85% of the world; TC^a^ value is 895 times, TC/P^b^ value is 19.04, the h-index value is 17; followed by the University of Tennessee Knoxville in the United States (36, 2.95%, 806, 22.39, 15). Among them, the Centre National De La Recherche Scientifique CNRS in France has a high TC/P^b^ value of 40.57, h-index. The value is also the highest at 18, showing that the Centre National De La Recherche Scientifique CNRS has a tremendous academic influence. As shown in Figure 3, the United States and China have the most frequent cooperation, followed by the United States and India and China and India. Among the top ten institutions publishing papers, five institutions (50%) belong to the United States, and two institutions (20%) belong to China, indicating that China and the United States have played a leading role in research in this field, and the United States is more influential. Washington State University is not only the most productive institution in the field but also the most core and cooperative institution in the field. It is worth noting that while India ranks third in the world for research publications in this field, India does not have a single institution in the top ten publications. It can be seen that some of the articles in India are only in quantity, and there are few articles of high quality and value journals.

#### 3.2.4 Cited-journal analysis

Related articles in the field of biodegradable films for soil have been published in a total of 200 journals in the Web of Science Core Collection (ScienceCitation Index Expanded, SCIE) database, and the top 10 journals with published papers (Table 3) and the number of citations Top 10 articles (Table 4). As can be seen from Table 3, JPolymer Degradation and Stability is the most published journal, with a total of 64 related articles, accounting for 5.24% of all articles, and the journal has a total of 27,899 citations; followed by Journal of Polymers and the Environment, published 62 related articles, accounting for 5.07% of all articles, and the journals were cited 6,899 times. Among the top ten journals with published articles, four journals belong to the United States, which shows that the United States has a very high research status in this field. It is worth noting that the top ten journals with published papers are all developed country journals. It can be seen that there are many types of research and articles on environmental protection, energy-saving, and consumption reduction in developed countries. In addition, the impact factor (IF) and JCI of these journals can also measure their value according to their role and status in science communication. Among them, “Carbohydrate Polymers” has the highest JCI of 1.99, which is 99% higher than the average citation impact of similar journals, and the IF value is 8.678, which indicates that the journal has significant international influence. There are seven journals whose citation impact (JCI > 1) and impact factor (IF > 4) are higher than the average citation impact of similar journals, and the remaining six journals are: “Polymer Degradation and Stability,” with a JCI value of 1.11, higher than similar journals The average value is 11%, and the IF value is 5.207; the JCI value of “Science of the Total Environment” is 1.66, which is 66% higher than the average cited the impact of similar journals, and the IF value is 7.842; the JCI value of “Agricultural Water Management” is 1.71. The citation impact is 71% higher than the average citation impact of similar journals, with an IF value of 5.12; the JCI value of the International Journal of Biological Macromolecules is 1.38, which is 38% higher than the average citation impact of similar journals, with an IF value of 6.737; the CI value of Waste Management The JCI value of International Biodeterioration & Biodegradation is 1.02, which is 2% higher than the average citation impact of similar journals, and the IF value is 4.719.

**TABLE 3:** Top ten most productive institutions in terms of relevant articles.

Institutions (biodegradable film for soil)	Ps	Percentage (%)	Country	TC^a^	TC/P^b^	h-index
Washington State University	47	3.85	United States	895	19.04	17
University of Tennessee Knoxville	36	2.95	United States	806	22.39	15
University of Tennessee System	36	2.95	United States	806	22.39	15
Northwest A&F University China	31	2.54	China	1,085	35	18
Chinese Academy of Sciences	29	2.37	China	336	11.59	11
UT Institute of Agriculture	29	2.37	United States	723	24.93	14
Centre National De La Recherche Scientifique CNRS	28	2.29	France	1,136	40.57	18
Consiglio Nazionale Delle Ricerche CNR	24	1.96	Italy	669	27.88	15
United States Department of Agriculture USDA	24	1.96	United States	939	39.13	16
Russian Academy of Sciences	19	1.55	Russia	276	14.53	10

**TABLE 4:** Top ten journals with publication volume.

Journal	Ps	Percentage (%)	Publisher	Region	Total citations	IF	JCI
Polymer Degradation and Stability	64	5.24	Elsevier SCI Ltd.	England	27,899	5.207	1.11
Journal of Polymers and the Environment	62	5.07	Springer	United States	6,899	3.536	0.6
Journal of Applied Polymer Science	41	3.36	Wiley	United States	68,877	2.754	0.7
Science of the Total Environment	29	2.37	Elsevier	Netherlands	210,144	7.842	1.66
Carbohydrate Polymers	22	1.80	Elsevier SCI Ltd.	England	104,570	8.678	1.99
Agricultural Water Management	21	1.72	Elsevier	Netherlands	22,091	5.12	1.71
Hortscience	20	1.64	Amer Soc Horticultural Science	United States	12,336	1.617	0.59
International Journal of Biological Macromolecules	19	1.55	Elsevier	Netherlands	79,247	6.737	1.38
Waste Management	18	1.47	Pergamon-Elsevier Science Ltd.	United States	41,731	7.907	1.16
International Biodeterioration & Biodegradation	17	1.39	Elsevier SCI Ltd.	England	14,127	4.719	1.02

Note:JCI:Journal Citation Indicator; IF: 5-year impact factor, impact factor data from the 2020 edition of Journal Citation Reports® in Web of Science. The Journal Citation Indicator (JCI) is the average Category Normalized Citation Impact (CNCI) of citable items (articles and reviews) published by a journal over a recent 3 year period. The average JCI in a category is 1. Journals with a JCI of 1.5 have 50% more citation impact than the average in that category.

As can be seen from Table 4, two articles in the field of biodegradable films for soil have been cited more than 1000 times, namely “Advanced oxidation processes for organic contaminant destruction based on the Fenton reaction and related chemistry” published in 2006 and “Polylactic acid technology” published in 2000. These two articles are the most representative and have particular reference values and foundations for researchers to engage in research in this field. Pignatello, JJ et al. (Pignatello et al., 2006) published “Advanced oxidation processes for organic contaminant destruction based on the Fenton reaction and related chemistry,” which mainly describes the complex mechanisms of Fenton and Fenton-like reactions and the crucial factors affecting these reactions, in water and soil Applications in processing, with sections devoted to non-classical pathways, by-products, kinetic and process modeling, experimental design methods, soil and aquifer processing, the use of Fenton in conjunction with other advanced oxidation processes or biodegradation. “Polylactic acid technology,” published by Drumright, RE, et al. (Drumright et al., 2000), mainly describes the polylactic acid technology that can replace petrochemical products. Because of its biodegradability as a renewable resource, it can be actively applied in film and packaging, increasing the need for demand for agricultural products such as corn and sugar beets and reducing plastics’ dependence on oil.

### 3.3 Field experiments with biodegradable films for soil

The difference between the plastic film and biodegradable film used for the soil on tomatoes was explored through field experiments. It was found that compared with bare soil, both of them could increase the soil temperature respectively, and the plastic film was more significant, and the plastic film increased. The soil temperature ranged from 5.9°C to 6.9°C, while the biodegradable film was 5.5°C–5.8°C. Compared with bare soil, the increase in temperature under the film will limit soil microbial activity. Compared with bare soil next to the crop, plastic film, Its soil microbial biomass carbon (SMBC) decreased from 1703 ng g^−1^ to a minimum of 1005 ng g^−1^, and its soil organic matter mineralization (SOMM) decreased from 558 μg OM g^−1^ to a minimum of 451 μg OM g^−1^ for 15 days^−1^; while the biodegradable film decreased to a minimum value of 1183 ng g^−1^ and 499 μg OM g^−1^ 15 days^−1^, respectively, showing that the negative impact of plastic film on soil microbial characteristics was more significant (Moreno and Moreno, 2008).

Due to plastic films, plastic residues may accumulate in the soil, which poses serious environmental problems for agroecosystems. As an alternative, biodegradable films are expected to reduce plastic debris accumulation and soil pollution. Yueling Qi et al. (Y. Qi Y et al., 2020) found that biodegradable plastic residues significantly affected rhizosphere bacterial communities and rhizosphere volatiles mixture. When harvested at 4 months, compared with the pH value of the blank control of 7.01, the pH values of the plastic film and the biodegradable thin film decreased, and the pH value of the plastic film decreased more significantly. The pH of Ma size and Mi size plastic films was 6.86 and 6.91, respectively, while the pH values of Ma-size and Mi-size biodegradable films were 6.91 and 6.96, respectively. Compared with the blank control C:N of 15.84, Mi-sized plastic film and Mi-sized biodegradable film are more significant, 19.43 and 18.84, respectively.

Biodegradable films are recommended as a viable option for conventional plastic films for winter rapeseed production. Xiao-Bo Gu et al. (Gu et al., 2017), a 3-year field experiment, systematically analyzed and compared conventional plastic films (PM) and biodegradable films (BM) and no plastic film filming (CK) on each index of winter rapeseed due to the degradation of biofilm, the increase of soil temperature and soil water storage in BM at 150 days after sowing was significantly lower than that of PM; The PM depth was 1.7 cm; the mean evapotranspiration (ET) in the BM group was 10.0% higher than that in the PM group and 10.4% lower than that in the CK group. BM’s average yield and water use efficiencies were 5.8% and 14.3% lower than those of PM and 38.4% and 54.5% higher than CK’s. The contents of erucic acid and glucosinolates in BM were lower than those in PM, while the contents of seed oil, protein, and oleic acid in BM and PM were not significantly different.

To determine the optimal biodegradable film for summer maize, Minhua Yin et al. (Yin et al., 2019) combined three biodegradable films (one with a fast degradation rate (BM1), one with a medium degradation rate (BM2), and one with a slow degradation rate (BM3)) were compared with plastic film (PM), and no film was used as a control (CK). Soil temperature and soil water storage increased significantly under BM1 (3.6°C higher at 5 cm and 2.4°C higher at 25 cm) and BM2 (3.7°C higher at 5 cm and 2.3°C higher at 25 cm) compared with CK before the biodegradable film degraded. The soil organic carbon of the CK group was 2.85%, 5.30%, 11.72%, and 15.07% higher than that of BM1, BM2, BM3, and PM, respectively; Average grain yield, water use efficiency, and net revenue in BM2 were 18.40%, 25.10% and 32.97%, 11.90%, 6.62% and 20.46%, 11.43%, 6.82%, and 15.71%, and 32.50%, 45.64% and 41.05% higher than in BM1, BM3, PM, and CK, respectively. The results show that, compared with BM1, BM3, and PM, the biodegradable film of BM2 can improve the soil environment, promote the growth of maize, increase the net income, and reduce the loss of soil organic carbon.

In order to verify the suitability of biodegradable films for peanuts to adjust soil temperature and moisture, improve photosynthesis and yield, Tao Sun et al. (T. Sun et al., 2018) combined four biodegradable films [0% (BM1), 10% (BM2), 15% (BM3) and 20% (BM4)], plastic film (PM), and no film (CK) were compared. The results showed that the soil temperature of the BM3 treatment was higher than that of the other three biodegradable films throughout the growing season. Compared with other treatments, BM3-covered peanuts maintained a higher leaf area index, chlorophyll content, and net photosynthetic rate in later growth stages.

Ning Chen et al. (Chen et al., 2020) investigated the effects of plastic film and biodegradable film on nitrogen absorption, distribution, and leaching in drip irrigation sandy land and found NO_3_-N concentration, N absorption, and NO_3_-N leaching under biodegradable film Unevenness increased with increasing nitrogen application rate. In the 0–20 cm soil layer, the NO_3_-N concentration of the biodegradable film increased by 3.9% compared with the plastic film. In arid areas, the biodegradable film can be used as a good substitute for plastic film to avoid plastic pollution, and In sandy farmland, the application of 210 kg ha^−1^ nitrogen fertilizer and the biodegradable film is the best solution.

Many scholars have compared plastic films for soil with biodegradable films in field trials, and appropriate film should be selected for different crops and geographical environments. However, the biodegradable film, with its superior characteristics and favorable factors for soil and plants, protects the ecological environment and biodiversity, maintains green and sustainable development, and the biodegradable film for soil has been obtained. More and more scholars supported and recommended and gradually entered the field of biodegradable films and carried out corresponding research.

### 3.4 Degradation process of biodegradable films

**FIGURE 4:**
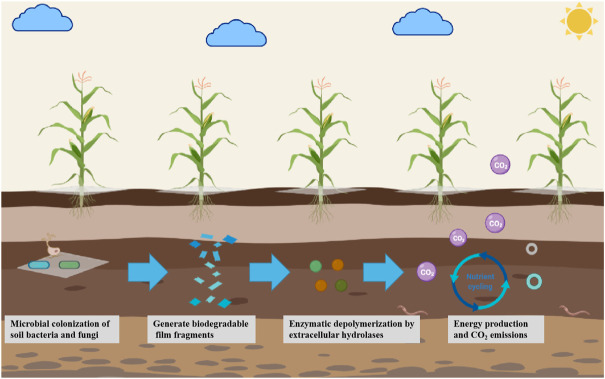
Main degradation processes of biodegradable films for soil.

The biodegradation process of biodegradable films used in the soil is mainly divided into three basic steps (Figure 4): 1) Microbial colonization of the surface of biodegradable films by bacteria and fungi in the soil. 2) The biodegradable film fragments undergo enzymatic depolymerization by extracellular hydrolases. Moreover, 3) Microorganisms absorb and utilize monomers and short oligomers to release CO_2_, generate energy, and promote the activity of soil microorganisms and the cycle of nutrients (Sander, 2019).

### 3.5 Analysis of the activity index and the Attractive Index

**FIGURE 5:**
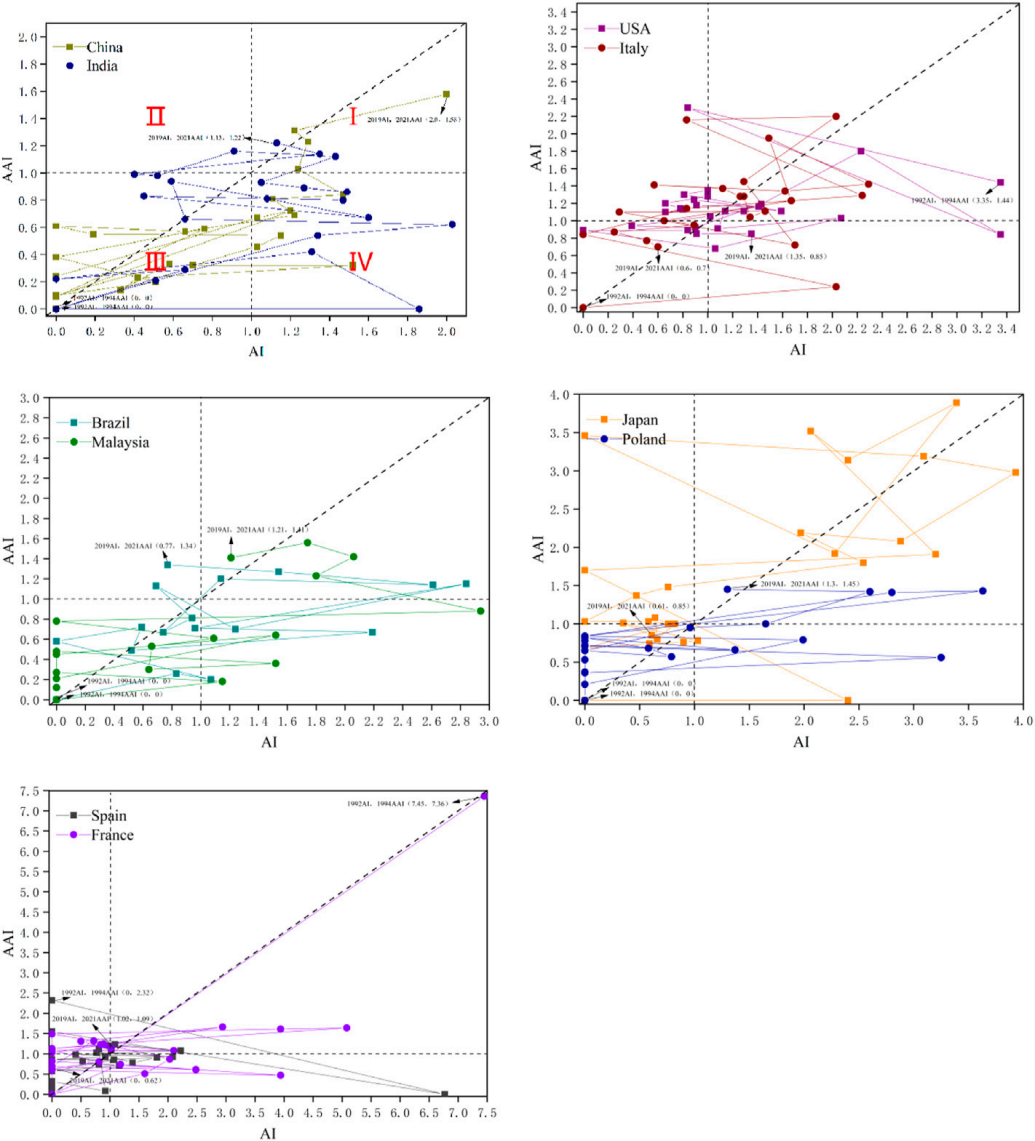
Relational chart of AI and AAI from 1992 to 2021 for 10 countries/regions.

In this part, we use the Activity Index (AI) and the Attractive Index (AAI) to evaluate the temporal changes in research and academic impact for selected countries. Considering there is usually a lag between when an article is published and cited, we set the time horizon for the attraction index to be 2 years later than the activity index. The starting year of the Activity Index for a given country is when the country first published an article in the field. Therefore, the activity and Attractive Index’s time horizons are set as 1992 to 2019 and 1994 to 2021, respectively. The Activity Index (AI) and Attractive Index (AAI) of the top 10 countries with the most publications were calculated (Supplementary Tables S1, S2) and presented in a quadrant diagram (Figure 5). The reference line (*y* = *x*) reflects how a country’s research efforts are balanced against the impact it cites in field research. These selected countries have undergone four types of evolution. Quadrants 1-4 in Figure 5 represent four different situations, respectively. The points in the first quadrant represent the years in which the country’s Activity Index (AI) and Attractive Index (AAI) are both higher than the global average; Dots represent years in which the country’s Attractive Index (AAI) is higher than the global average, and the Activity Index (AI) is lower than the global average; the dots in the third quadrant represent the country’s Activity Index (AI) and Attractive Index (AAI) are both low Years in which the country’s Activity Index (AI) is above the global average. The Attractive Index (AAI) is below the global average.

Comparing country performance using the Activity Index (AI) and the Attractive Index (AAI) shows that, in most years, the United States, Italy, Japan, and France have higher research effort and academic influence than the global average; China, India, Malaysia, Brazil Research effort and academic impact in, Poland and Spain are below the global average in most years. But developing countries (China, India, Malaysia, Poland, and Brazil) have surpassed the global average since 2019, which means research, technology, and economies in developing countries are starting to improve; developed countries (US, Italy, Japan, France and Spain) research effort and impact declined relatively over the same period. However, developed countries still significantly influence and status on the research of biodegradable films for soil. Furthermore, China and Poland are relatively close to the reference line in terms of the distance between the points representing a particular country and the reference line, which means that their research efforts are balanced against the impact of citations.

### 3.6 Keywords in different countries

**FIGURE 6:**
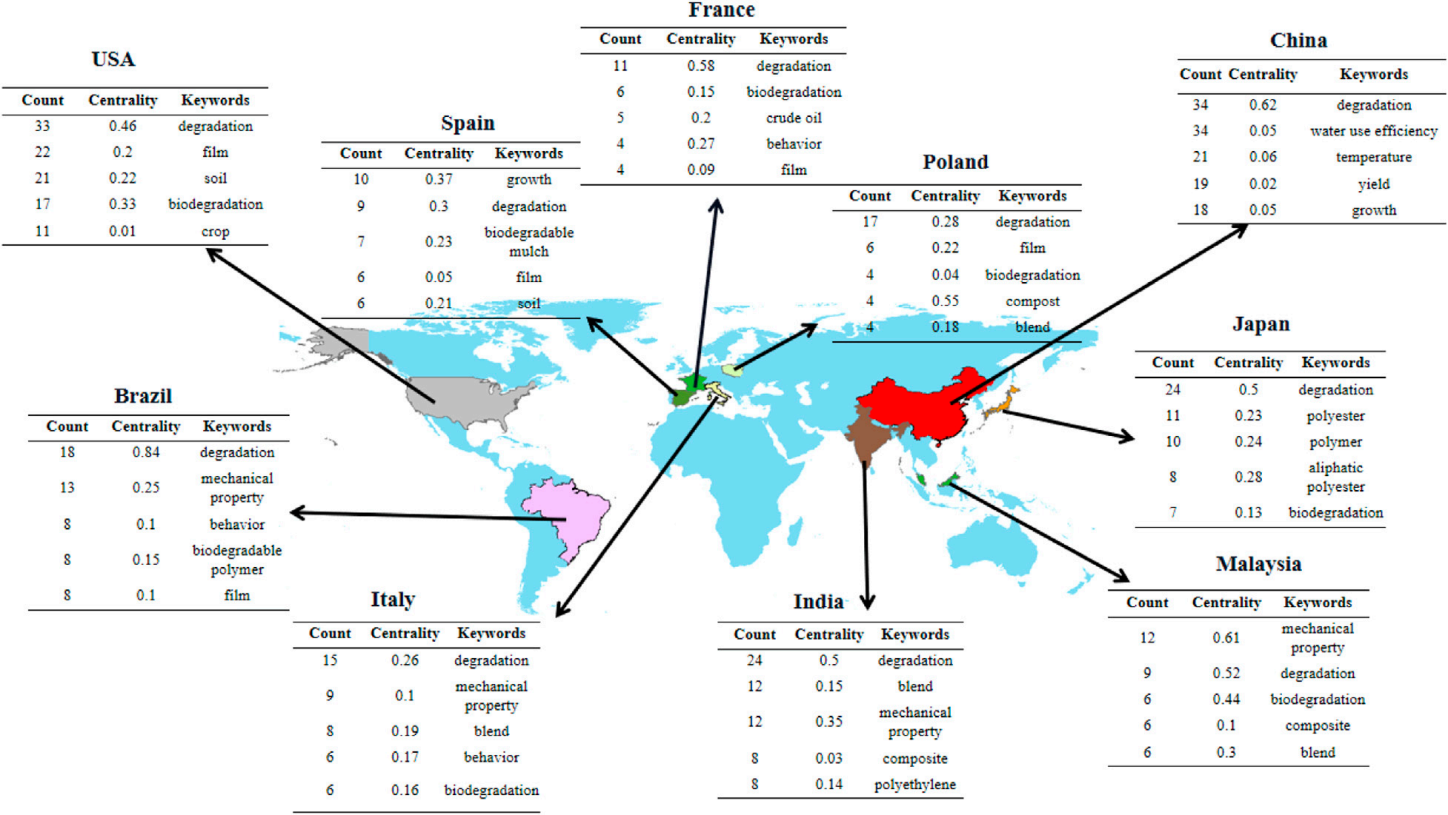
The 10 most frequently used keywords in the 10 most influential countries. Note: In the picture, China includes mainland China only.

This field has been extensively studied worldwide based on biodegradable films for soil. However, the subjects studied vary from region to region due to differences in geographical features, history, and economic and climatic conditions. Figure 6 provides the five most frequently used keywords in the ten most influential countries. Keywords vary from country to country. For example, from 1992 to 2021, the most frequently used keywords in China are “degradation” (34 times, centrality is 0.62), “water use efficiency” (34 times, centrality is 0.05), “temperature” (21 times, centrality 0.06), while the most frequently used keywords in the United States are “degradation” (33 times, centrality 0.46), “film” (22 times, centrality 0.2), and “soil” (21 times, centrality 0.22). It is worth noting that, as seen from Figure 6, among the keywords of 10 countries, the betweenness centrality of the keyword “degradation” is more significant than 0.1, which shows that it is used as a critical node in different countries. Other keywords play an excellent role in mediating connections.

### 3.7 Knowledge structure and sub-fields

**FIGURE 7:**
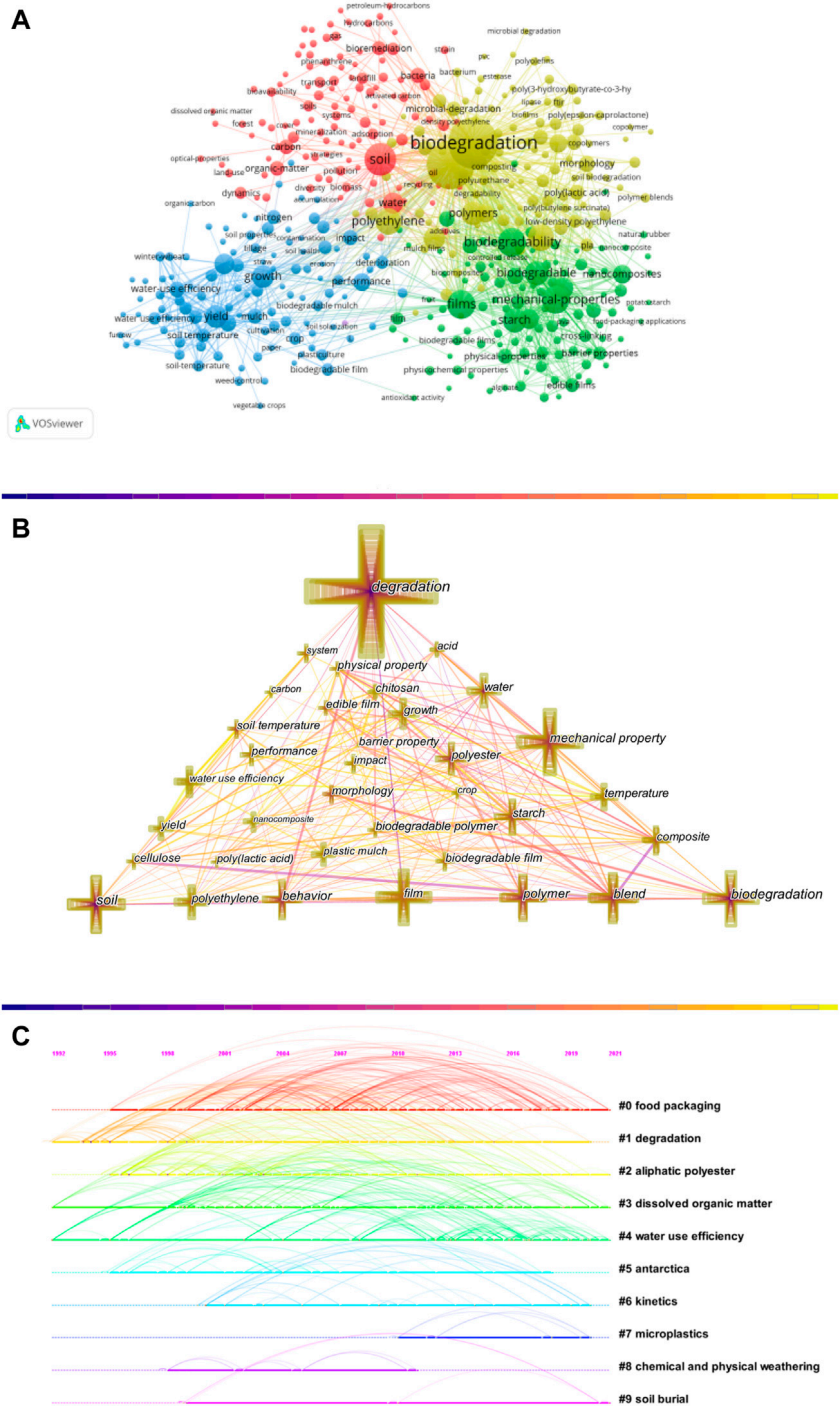
Keyword co-occurrence and clustering knowledge graph.**(A)**Based on VOS Viewer software.**(B)**Based on CiteSpace software.**(C)**Based on CiteSpace software.

As the core of a document, keywords are words with substantive meaning condensed from the document’s leading content and have a high guiding role for academic research in a field. Keyword co-occurrence is to mine the relationship between high-frequency keywords. If a particular keyword appears in different documents with high frequency simultaneously, their correlation is very close, representing the hot research in this field. Only considering the relationship of the keyword dimension for clustering has certain limitations, ignoring the influence of cooperative relationships (such as countries, authors, institutions, and journals), and it is not easy to deeply interpret the Frontier fields in terms of classification. Therefore, this study constructed a bimodal matrix, that is, systematic clustering analysis of high-frequency keywords. Citespace software has diversified, time-sharing, and dynamic citation visualization analysis, allowing readers to understand a topic’s knowledge domain through visualization (C. M. Chen et al., 2010; Xie, 2015). The keywords in the field of biodegradable films for soil were visualized and analyzed by VOS Viewer software (Figure 7A); Visualize the data to draw a graph of keyword co-occurrence (Figure 7B) and a graph of keyword clusters (Figure 7C). In Figure 7A, blue, red, green, and yellow represent four clusters; the color of the section in Figure 7B represents the year, the size of the node represents the number or frequency, the larger the node, the more the number or frequency; The line between them represents the strength of the association, and the color of the line represents the change in the years of cooperation. In addition, the thicker the node ring, the higher the frequency of terms, and the thicker the line between nodes, the closer the distance between them (Ping et al., 2017); Figure 7C performs a top ten systematic clustering of all related keywords.

As can be seen from Figure 7A, the keywords are clustered into 4 clusters, which are the yellow clusters represented by “biodegradation,” “polymers,” and “polyethylene”; the yellow clusters represented by “soi,” “water,” and “bacteria” Red clusters; green clusters represented by “biodegradability,” “films” and “starch”; and blue clusters represented by “growth,” “yield” and “film.” It can be seen from Figure 7B that the node with the keyword “degradation” is the most significant, indicating the highest frequency of occurrence, followed by the keywords “mechanical property,” “soil,” “biodegradation,” “film,” and “blend.” Through keyword clustering, the keywords are systematically clustered into 10 clusters such as “food packaging,” “degradation,” and “aliphatic polyester."Using CiteSpace software, 1222 articles about the field of biodegradable films for soil were analyzed by time-segment keyword, as shown in Table 5. The keyword “degradation,” the most critical node, has the most significant number and the highest betweenness centrality in the first two periods. It is worth noting that although the number of this node has been increasing in the three periods, the betweenness centrality is decreased from 0.45 in 1992–2001 to 0.18 in 2012–2021, indicating that its ability to act as a medium began to decline; at the same time, the betweenness centrality of the keyword “biodegradation” reached 2012–2021. The highest value is 0.19, which exceeds “degradation.” It can be seen that the research on biodegradation has gradually replaced the research on degradation, and biodegradation will continue to be a hot research direction for researchers in the future.

**TABLE 5:** Top ten cited articles and journals.

Title	Journal	Citations	Citations per year	Year	First author
Advanced oxidation processes for organic contaminant destruction based on the Fenton reaction and related chemistry	Critical Reviews in Environmental Science and Technology	2,534	158.38	2006	Pignatello, JJ (Pignatello et al., 2006)
Polylactic acid technology	Advanced Materials	1,946	88.45	2000	Drumright, RE (Drumright et al., 2000)
Polyethylene and biodegradable films for agricultural applications: a review	Agronomy for Sustainable Development	511	51.10	2012	Kasirajan, S (Kasirajan and Ngouajio, 2012)
‘White revolution’ to ‘white pollution'-agricultural plastic film film in China	Environmental Research Letters	280	35.00	2014	Liu, EK (Liu et al., 2014)
Municipal solid waste management from a systems perspective	Journal of Cleaner Production	272	16.00	2005	Eriksson, O (Eriksson et al., 2005)
Environmental stability of selected petroleum hydrocarbon source and weathering ratios	Environmental Science & Technology	265	10.19	1996	Douglas, GS (Douglas et al., 1996)
Mechanical, barrier, and biodegradability properties of bagasse cellulose whiskers reinforced natural rubber nanocomposites	Industrial Crops and Products	251	20.92	2010	Bras, J (Bras et al., 2010)
Macro- and micro- plastics in soil-plant system: Effects of plastic film film residues on wheat (*Triticum aestivum*) growth	Science of the Total Environment	243	60.75	2018	Qi, YL (Qi et al., 2018)
Wood-Based Nanotechnologies toward Sustainability	Advanced Materials	226	56.50	2018	Jiang, F (Jiang et al., 2018)
Biodegradation behavior of poly (butylene adipate-co-terephthalate) (PBAT), poly (lactic acid) (PLA), and their blend under soil conditions	Polymer Testing	223	24.78	2013	Weng, YX (Weng et al., 2013)

**FIGURE 8:**
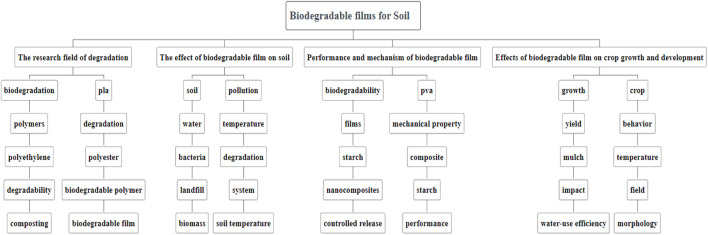
A map of knowledge structures for biodegradable film for soil.

In summary, the research on biodegradable membranes for soil can be divided into four main categories of knowledge structures (Figure 8): The research field of degradation, The effect of biodegradable film on soil, the Performance and mechanism of biodegradable film, and the Effects of biodegradable film on crop growth and development.

#### 3.7.1 The research field of degradation

Synthetic polymers are mainly derived from petrochemical raw materials (crude oil, natural gas) and have a very long period of self-degradation, which can last for decades or even hundreds of years. According to statistics, synthetic polymers account for nearly 98% of the polymer materials currently produced, of which more than 80% are produced by petrochemicals (Song et al., 2009; Galizia and Bye, 2018). Plastics are resistant to microorganisms, and due to their short existence in natural evolution, it is impossible to design new enzymatic structures capable of degrading synthetic polymers (Mueller, 2006). The dramatic increase in production and lack of biodegradability of some polymers, such as plastic films for packaging, industry, agriculture, etc., has drawn public attention to the enormous environmental accumulation and pollution problems that may persist for decades or even centuries (Malizia and Monmany-Garzia, 2019). Biodegradable materials can degrade and have faster degradation rates in municipal and industrial biological waste treatment facilities, opening the way for the development and innovation of waste management strategies (Witt et al., 1997; Raddadi and Fava, 2019). Therefore, the research and development, promotion, and use of biodegradable materials have become the general trend in the future.

#### 3.7.2 The effect of biodegradable film on soil

Nowadays, biodegradable materials are gradually accepted by society, and after they are used in soil, they can be decomposed by bacteria in the soil environment (Purahong et al., 2021; Tanunchai et al., 2021). However, the impact of these natural degradation processes on soil bacteria is unclear, and it is feared that the decomposition of the biodegradable film by bacteria will disrupt the soil bacterial community structure (Breza-Boruta et al., 2016). Several studies have shown that biodegradable film results in increased microbial biomass and enzymatic activity in soil (Bonanomi et al., 2008; Yamamoto-Tamura et al., 2015), increased soil fungal abundance (Rychter et al., 2006; Ma et al., 2016), and altered soil microbial community structure (C. H. Li et al., 2014; Muroi et al., 2016), ultimately altering carbon cycling and storage in soils. Masui A et al. (Masui et al., 2011)proved that if the biodegradable film is used in the farmland in the usual way and frequency and then plowed into the farmland, there is almost no pressure on the soil environment, but it has a significant impact on the soil environment such as the soil bacterial community. Rong Li et al. (R. Li et al., 2016)used polyethylene film (PM), biodegradable polymer film (BM), corn stover (MM), liquid film (LM), and uncovered film in a field experiment on the Loess Plateau of China over 3 years. Comparing the control (CK), five film treatments verified that the biodegradable film could significantly improve the soil moisture and temperature conditions in the semi-arid Loess Plateau and reduce soil environmental pollution compared with other treatments. Biodegradable film fragments added to soil are generally considered safe from a toxicological standpoint. For example, Sforzini S et al. (Sforzini et al., 2016) tested a starch copolyester blend, Mater-Bi R, showing no ecotoxicity effects, and Ardisson GB et al. (Ardisson et al., 2014)verified experimentally that biodegradable film fragments did not negatively affect nitrification potential effect.

#### 3.7.3 Performance and mechanism of biodegradable film

The biodegradable film is a biodegradable aliphatic-aromatic copolymer with excellent tensile strength and toughness (Raquez et al., 2008). Although the degradation of these biodegradable films facilitates their export management, the various properties of the films (water vapor permeability, mechanical and radiation properties) also gradually decline over time (Briassoulis, 2007; Bilck et al., 2010). In addition to self-aging, degradable thin films undergo biodegradation mechanisms by soil bacteria (W. Wang et al., 2021), fungi (Kessler et al., 2014), microbial enzymes (Bhardwaj et al., 2013), and other factors (animals, earthworms, ants). Biodegradable films can also be blended with biodegradable polymers to improve processability and mechanical properties. Touchaleaume F et al. (Touchaleaume et al., 2018), by comparing the field experiments of biodegradable film mixtures (PBAT/PLA, PBAT/PPC, and PBAT/starch), verified the degradation status of the biodegradable film and the location of the material, soil burial The different aging mechanisms occurred at the site and the upper part of the soil, and although the mechanical properties decreased due to factors such as ultraviolet rays, the biodegradability of the material did not change even after 18 months of field aging (Xiang et al., 2021), verified that the inhibitory effect of heavy soil metals on biodegradability combined with three-dimensional cross-linked superabsorbent polymers can effectively reduce the nutrient release of the biodegradable film. Javadi et al. (Javadi et al., 2010), blended the polyhydroxyalkanoate copolymer poly (3-hydroxybutyrate-co-3-hydroxy valerate) with a biodegradable film by microporous and conventional injection molding, significantly improving its toughness. Biodegradable materials can be combined with organic materials, such as food, vegetable waste, and manure, to generate carbon-rich composts (Mujtaba et al., 2021), which must maintain their physical and mechanical properties at the time of use, but also at the end of use. Composted or biodegraded by microorganisms (Abu Qdais and Hamoda, 2004). Most ecotoxicity studies have been performed after the biodegradable film has degraded in soil or compost (Kapanen et al., 2008; Ardisson et al., 2014). In contrast, the biodegradable film may be abiotically degraded in contact with the natural environment before entering soil (such as hydrolysis) (Kapanen et al., 2008). H. Serrano-Ruíz et al. (Serrano-Ruiz et al., 2020) found that BDP releases compounds in the aqueous environment before biodegradation begins. Fertilizer coating is a general controlled release method. The coating material insulates the fertilizer from the soil and slowly dissolves into the soil—the role of proportionality (Ge et al., 2002). Lu P et al. (Lu et al., 2016) studies have shown that polymer-coated urea (PCU), a new type of controlled-release fertilizer, is biodegradable in soil compared to conventional starch coatings and is more efficient in water and soil. They have a reduced nitrogen release rate. For some dry regions worldwide, superabsorbent polymers as coating materials can add moisture retention to fertilizers (Bao et al., 2015). Sarmah D et al. (Sarmah and Karak, 2020)conducted experiments on the superabsorbent hydrogel of starch-modified polyacrylic acid and found that it can be used as a controlled release carrier and water retention agent in different fields.

#### 3.7.4 Effects of biodegradable film on crop growth and development

Covering the soil with the plastic film will promote the growth and development of annual and perennial crops and increase the yield. Film can effectively improve the field microclimate for crop growth, increase crop yield, and enable crops to develop well in soils with little water (Lamont, 2005; R. Li R et al., 2021). Experiments have shown that the application of biodegradable film in the soil can promote the growth and development of crops such as pepper (Mari et al., 2020), tomato (Cirujeda et al., 2012; Abduwaiti et al., 2021), corn (N. Chen et al., 2021), and cotton (L. X. Shen et al., 2019; Z. H. Wang et al., 2019). The utilization rate of water is conducive to increasing temperature and moisture, promoting the increase of output and bringing more excellent economic benefits. Biodegradable film can eventually be integrated directly into the soil, where soil microbes convert carbon dioxide or methane, water, and biomass for uptake by some crops (Immirzi et al., 2003). Caruso G et al. (Caruso et al., 2019) validated that the use of biodegradable film in soil resulted in a 17.4% higher Phytoanalytical Development (SPAD) index of growing leaves and a significant improvement in Perennial wall rockets’ leaf quality.

### 3.8 Research Frontier identification

Using CiteSpace software for explosive detection of literature, Table 6 shows the top 25 explosive keywords. From the most intense keywords, we can determine the development of keywords and potential research topics (Y. N. Li Y. N et al., 2021), which can be clearly defined. See when keywords appear, when they end, keyword strength, and predict future research trends in hot areas. As shown in Table 6, the earliest and longest-lasting keyword was “bacteria” in 1998, with a burst intensity of 3.74, lasting 17 years. The keyword “poly (3 hydroxybutyrates)” had the highest burst strength of 5.63. In addition, we can see that 8 explosive keywords such as “impact”, “nanocomposite”, “quality”, “water use efficiency”, “crop”, “moisture”, “chitosan” and “carbon” have been emerging in recent years. It will continue to explode in 2021, so it can be speculated that these keywords will still become the hot research direction of scholars and institutions in this field. From these eight keywords, it can be concluded that in the future, scholars and institutions in the field of biodegradable soil films will shift from single research to more diversified research, focusing on the ecological and environmental protection of biodegradable films, crop growth, and development, and research on soil traits.

**TABLE 6:** Related keywords of biodegradable films for soil in different periods from 1992 to 2021.

1992–2001			2002–2011			2012–2021		
Keywords	Ps	Centrality	Keywords	Ps	Centrality	Keywords	Ps	Centrality
degradation	16	0.45	degradation	62	0.41	degradation	158	0.18
biodegradation	7	0.17	biodegradation	27	0.28	film	72	0.07
soil	6	0.11	polymer	25	0.1	mechanical property	71	0.08
blend	5	0.1	mechanical property	23	0.14	soil	65	0.14
polymer	4	0.06	soil	22	0.23	biodegradation	54	0.19
polyvinyl alcohol	4	0.02	behavior	19	0.13	blend	54	0.08
copolymer	3	0.04	blend	18	0.13	growth	45	0.05
microorganism	3	0.09	poly (3 hydroxybutyrate)	11	0.03	polymer	40	0.07
biodegradability	3	0.02	water	9	0.02	yield	40	0.05
film	3	0.03	biodegradable polymer	9	0.05	water	39	0.13

Combined with the analysis results of keyword clustering analysis, the emerging research fronts of biodegradable films for soil are summarized as follows: removal and disposal of traditional polyethylene film remain significant agronomic, economic, and environmental constraints; biodegradable plastic films transform soil carbon dioxide, water, and natural matter through microbial activity, contributing to microclimate change, soil biota, soil fertility, and crop yield. It has positive effects; biodegradable films are widely used in agriculture to increase crop yield, soil moisture, and increase soil temperature by suppressing weeds and protecting crops; microplastics may interact with soil physicochemical properties and organisms, negatively affecting plant growth Impact, to mitigate environmental plastic pollution, biodegradable films are replacing non-biodegradable polymers; excess plastic residue soils lead to soil pollution, vegetation destruction in horticulture and agriculture. Biodegradable films are considered ideal substitutes for traditional plastics due to their biodegradable properties to reduce plastic waste and promote ecologically sustainable development; chitosan, as new film material, can be exceptional. The biodegradability of the enhanced film, its co-use with other materials, and its contribution to the ecological soil environment system needs to be further studied. Table 7.

**TABLE 7:** Top 20 keywords with strongest citation bursts.

Keywords	Year	Strength	Begin	End	1992–2021
bacteria	1992	3.74	1998	2014	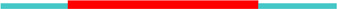
polymer	1992	4.48	2001	2012	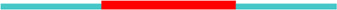
poly (3 hydroxybutyrate)	1992	5.63	2003	2012	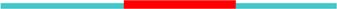
behavior	1992	4.21	2006	2011	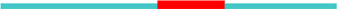
low density polyethylene	1992	3.77	2007	2012	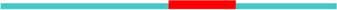
morphology	1992	4.53	2009	2018	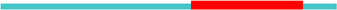
ldpe	1992	3.67	2009	2015	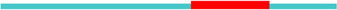
polyester	1992	4.48	2010	2014	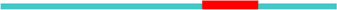
thermal degradation	1992	3.56	2011	2012	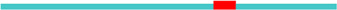
wheat	1992	4.43	2012	2017	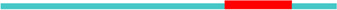
soil	1992	3.52	2012	2014	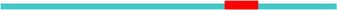
impact	1992	3.65	2016	2021	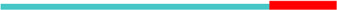
food packaging	1992	3.79	2017	2019	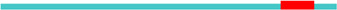
nanocomposite	1992	3.87	2018	2021	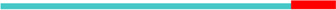
quality	1992	3.74	2018	2021	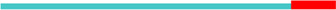
water use efficiency	1992	4.77	2019	2021	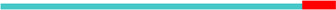
crop	1992	4.7	2019	2021	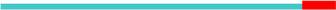
moisture	1992	4.01	2019	2021	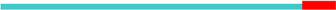
chitosan	1992	3.98	2019	2021	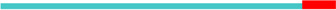
carbon	1992	3.78	2019	2021	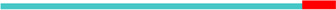

## 4 Conclusion

This paper presents a retrospective evaluation of the field of biodegradable films used in soils over the past three decades from a bibliometric perspective. Through quantitative analysis and network mapping, we identified the most influential subject categories/countries/institutions/journals, understood collaborative networks, and visualized the evolution of keyword co-occurrence networks.

Many scholars have compared the best choices of different plants for soil plastic films and biodegradable films in field experiments. The results show that biodegradable soil films are supported and recommended by more and more scholars due to their superior characteristics. Moreover, it gradually entered the field of biodegradable films and carried out corresponding research.

Comparing country performance using the Activity Index (AI) and Attractive Index (AAI) shows that developing countries (China, India, Malaysia, Poland, and Brazil) have surpassed the global average since 2019, meaning that research in developing countries, technology, and the economy began to improve; however, developed countries still have a significant influence and position in the research of biodegradable films for soil. Furthermore, China and Poland are relatively close to the reference line in terms of the distance between the points representing a particular country and the reference line, which means that their research efforts are balanced against the impact of citations.

As the concepts of environmental protection, energy saving, low carbon, emission reduction, and sustainable development are highly praised worldwide, the research on biodegradable films will continue to increase. Transforming biodegradable membranes will become a research hotspot and research trend for membranes used in soil, and future research trends in this field will be diversified in many countries. Scholars and institutions engaged in biodegradable soil film will shift from single research to diversified research, focusing on the research field of degradation, the effect of biodegradable film on soil, performance, and mechanism of biodegradable film, and the effects of biodegradable film on crop growth and development.

This study can be seen as a microcosm of the field of biodegradable soil membranes, and it will help researchers quickly identify their general patterns. Readers can gain exciting information from the rich bibliometric data. There are still some limitations of this study; the data in this paper are only from the Web of Science Core Collection (ScienceCitation Index Expanded, SCIE) database and may not include other citation information. Despite the above limitations, this paper can provide suggestions for scholars interested in identity recognition to help them understand development trajectories and statistical models more quickly.

## Data Availability

The original contributions presented in the study are included in the article/Supplementary Material, further inquiries can be directed to the corresponding author.
