# Supplementary material for: Past, present, and future perspectives of biodegradable films for soil: A 30-year systematic review
Source: Front Bioeng Biotechnol. 2022 Oct 17;10:1006388. doi: 10.3389/fbioe.2022.1006388 (PMC9621393; doi:10.3389/fbioe.2022.1006388)
Supplement: Supplementary file 1 [file DataSheet1.PDF]

Supplementary Material

Table S1. The AI index for 10 countries

AI	China	USA	India	Italy	Brazil	Japan	Spain	France	Malaysia	Poland
1992	0.00	3.35	0.00	0.00	0.00	0.00	0.00	7.45	0.00	0.00
1993	0.00	0.84	0.00	0.00	0.00	2.40	6.77	0.00	0.00	0.00
1994	0.00	3.35	0.00	0.00	0.00	0.00	0.00	0.00	0.00	0.00
1995	0.00	2.23	0.00	0.00	0.00	3.20	0.00	2.48	0.00	0.00
1996	1.03	1.43	0.00	2.03	0.00	2.06	0.00	0.00	0.00	0.00
1997	1.20	0.84	1.86	0.00	0.00	2.40	0.00	0.00	0.00	0.00
1998	0.00	0.00	1.31	0.83	0.00	3.39	0.00	3.94	0.00	0.00
1999	0.76	1.06	0.00	2.24	0.00	2.28	0.00	1.18	0.00	3.25
2000	0.00	2.07	0.66	0.83	0.00	0.00	0.00	0.00	0.00	0.00
2001	0.51	1.08	0.00	2.03	0.00	3.09	0.00	1.60	0.00	0.00
2002	0.00	1.14	0.51	1.29	0.00	3.93	0.92	2.03	0.00	0.00
2003	0.58	1.41	1.34	1.70	0.00	2.88	0.81	0.89	1.15	0.00
2004	0.33	0.91	2.03	0.65	0.83	1.97	0.92	5.08	0.00	2.80
2005	0.42	0.89	0.66	1.67	1.07	2.54	0.00	3.94	0.00	3.63
2006	1.52	1.59	0.59	1.49	0.00	0.76	1.07	0.00	1.52	0.00
2007	0.70	0.81	1.08	2.29	0.59	0.47	0.00	0.72	0.00	1.99
2008	1.03	1.00	1.60	1.62	0.52	0.00	1.16	0.00	0.00	0.00
2009	1.15	1.00	0.45	0.57	2.19	0.58	0.81	0.00	0.00	0.00
2010	0.19	0.66	1.47	1.12	0.96	0.76	0.53	2.94	1.52	0.00

2011	0.00	0.78	1.49	1.26	2.84	0.64	1.81	0.50	0.64	1.37
2012	0.66	0.80	1.27	1.29	1.24	0.66	1.39	1.02	0.66	0.00
2013	1.22	0.66	1.05	1.34	0.69	0.82	0.77	0.84	1.09	0.58
2014	1.11	1.29	1.43	1.46	0.94	0.00	2.08	0.00	0.00	0.79
2015	1.47	1.02	0.91	0.29	0.74	0.59	0.41	0.91	2.94	0.00
2016	1.24	0.39	0.52	0.89	1.14	0.90	2.22	2.10	1.80	0.96
2017	1.29	0.90	0.40	0.25	2.61	1.03	1.09	0.00	2.06	1.65
2018	1.22	0.91	1.35	0.51	1.54	0.35	0.98	0.81	1.74	2.60
2019	2.00	1.35	1.13	0.60	0.77	0.61	1.02	0.00	1.21	1.30

Table S2. The AAI index for 10 countries

AAI	China	USA	India	Italy	Brazil	Japan	Spain	France	Malaysia	Poland
1994	0.00	1.44	0.00	0.00	0.00	0.00	2.32	7.36	0.00	0.00
1995	0.00	2.30	0.00	0.00	0.00	0.00	0.00	0.00	0.00	0.00
1996	0.00	0.84	0.00	0.00	0.00	1.70	1.55	0.82	0.00	0.00
1997	0.24	1.80	0.00	0.00	0.00	1.91	0.00	0.61	0.00	0.00
1998	0.67	1.19	0.00	0.24	0.00	3.52	0.00	0.68	0.00	0.00
1999	0.72	0.89	0.00	0.84	0.00	3.14	0.00	0.60	0.00	0.00
2000	0.09	0.89	0.42	1.14	0.00	3.89	0.22	0.47	0.00	0.71
2001	0.59	0.68	0.22	1.29	0.00	1.92	0.18	0.74	0.00	0.56
2002	0.38	1.03	0.29	2.16	0.00	3.46	0.11	0.00	0.00	0.36
2003	0.20	0.91	0.00	2.20	0.00	3.19	0.32	0.51	0.00	0.78
2004	0.10	1.11	0.21	1.45	0.00	2.98	0.08	0.87	0.00	0.53
2005	0.33	1.16	0.54	0.72	0.00	2.08	1.11	1.24	0.18	0.84
2006	0.14	1.18	0.62	1.00	0.26	2.19	0.92	1.64	0.27	1.41
2007	0.23	1.24	0.66	1.23	0.20	1.80	0.86	1.61	0.21	1.43
2008	0.32	1.11	0.94	1.95	0.58	1.48	0.85	1.49	0.64	0.84
2009	0.32	1.30	0.81	1.42	0.72	1.37	0.57	1.32	0.45	0.79
2010	0.46	1.35	0.67	1.34	0.49	1.03	0.71	0.70	0.12	0.21
2011	0.54	1.28	0.83	1.41	0.67	1.03	0.73	1.03	0.48	0.37
2012	0.55	1.20	0.80	1.37	0.71	1.00	0.82	1.66	0.36	0.72
2013	0.61	1.14	0.86	1.28	1.15	1.08	0.91	1.31	0.30	0.66
2014	0.57	1.13	0.89	1.28	0.70	0.80	0.78	1.12	0.53	0.82

2015	0.69	1.10	0.93	1.04	1.13	1.00	1.03	1.23	0.61	0.68
2016	0.81	1.11	1.12	1.11	0.81	0.68	0.93	1.13	0.78	0.57
2017	0.84	1.05	1.16	1.10	0.67	0.74	0.98	1.22	0.88	0.65
2018	1.03	0.94	0.98	0.95	1.20	0.76	1.08	1.08	1.23	0.95
2019	1.23	0.92	0.99	0.87	1.14	0.78	1.23	1.09	1.42	1.00
2020	1.31	0.85	1.14	0.77	1.27	1.01	1.20	0.80	1.56	1.42
2021	1.58	0.85	1.22	0.70	1.34	0.85	1.09	0.62	1.41	1.45
